# Use of a Novel Polymer-Coated Steel as an Alternative to Traditional Can Manufacturing in the Food Industry †

**DOI:** 10.3390/polym13020222

**Published:** 2021-01-11

**Authors:** Miguel A. Selles, Steven R. Schmid, Samuel Sanchez-Caballero, Maziar Ramezani, Elena Perez-Bernabeu

**Affiliations:** 1 Department of Mechanical and Materials Engineering, Universitat Politècnica de València, 03801 Alcoy, Spain; sasanca@dimm.upv.es; 2 Department of Mechanical Engineering and Engineering Science, University of North Carolina, Charlotte, NC 28223, USA; steve.schmid@uncc.edu; 3 Department of Mechanical Engineering, Auckland University of Technology, Auckland, WZ 1010, New Zealand; maziar.ramezani@aut.ac.nz; 4 Department of Statistics and Operations Research, Universitat Politècnica de València, 03801 Alcoy, Spain; elenapb@eio.upv.es

**Keywords:** coating, ironing, VOC, upper bound, polymer, coating, can, artificial neural network (ANN), friction, wear

## Abstract

Metal containers (both food and beverage cans) are made from huge steel or aluminum coils that are transformed into two- or three-piece products. During the manufacturing process, the metal is sprayed on both sides and the aerosol acts as insulation, but unfortunately produces volatile organic compounds (VOCs). The present work presents a different way to manufacture these containers using a novel prelaminated two-layer polymer steel. It was experimentally possible to verify that the material survives all the involved manufacturing processes. Thus tests were carried out in an ironing simulator to measure roughness, friction coefficient and surface quality. In addition, two theoretical ironing models were developed: upper bound model and artificial neural network. These models are useful for packaging designers and manufacturers.

## 1. Introduction

According to recent studies, nearly 100 billion food cans and 230 billion beverage cans are fabricated worldwide every year [1,2]. These figures are continually increasing, especially with rapidly expanding markets in Asia, the Middle East, and South America. Given high production volumes and fierce competition among can manufacturers, producers need to very accurately calculate the total cost of a single can. Even the slightest rise or drop in the cost of a single can as a result of a manufacturing change can strongly impact the economy for can makers [3].

The metal cans for beverages manufactured in Europe and Asia are typically 45% aluminum and 55% steel alloy, whereas practically all cans in the U.S.A. are made entirely of aluminum, although food containers are manufactured from steel stock [4].

**Figure 1:**
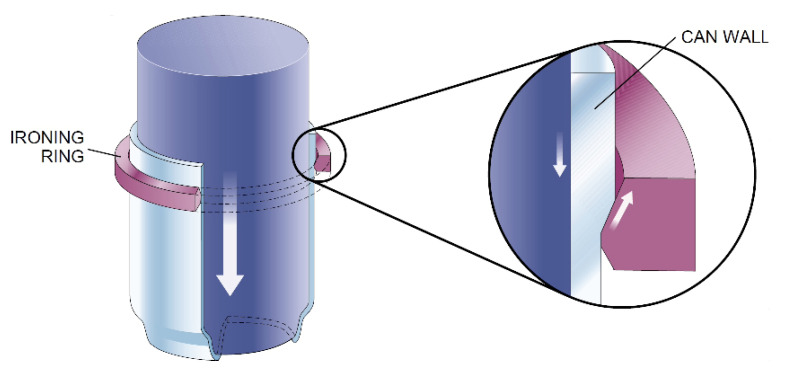
The ironing process in can manufacture. Source: Hosford and Duncan, Scientific American [5].

There are several processes involved to transform coil made of metal into cans: blanking, ironing, deep drawing, seaming, doming, necking, redrawing. Of all these processes, ironing is perhaps the most critical operation while forming the can body because of extremely high pressures, strains and strain rates associated with this manufacturing step. The punch, which is precisely dimensioned, holds and pushes the cup through two or three carbide ironing rings (Figure 1). Punch speed is faster than that of metal through the ironing zone, which thus causes the desirable elongation and thinning of cans during the process. Metal thickness is greater than the clearance between each ring and the punch. The punch surface generates friction that subsequently facilitates metal being pushed through ironing rings.

To remove any residual toxic lubricants and to apply a thin layer of polymer to base metal, cans are cleaned with water between the doming and necking processes. A spray is applied to cans to ensure a high-quality surface for can contents. However, when the substances in this spray are boiled, volatile organic compounds (VOC) may result due to the presence of polymer resins like methyl ethyl ketone. These resulting VOCs imply numerous health and environmental concerns, and one purpose of the canning industry is to eliminate VOCs during the production process [3].

Thermoplastic precoated rolled steels are a proven suitable alternative to base stocks in the previously described can manufacturing process. These steels are heated first, and then compacted between some polymer sheets. After this step, the resulting sheets are quenched to provide a very strong bond between the steel base and polymeric layers. These new coated steel types were studied by Jaworski et al. [6] and Huang et al. [7], and proved to be perfect materials for the forming process if some variables were carefully controlled. Polymer layers also offer the advantage of serving as solid lubricants and have the potential to remove VOCs during the manufacturing process.

A new polymer-coated steel with many layers, developed for use in food and beverage industries, extends previously conducted research on polymer-coated steels during forming processes [3,8]. Javorski and van der AA focused their research on the same material: PET coated steel. They found that with low die angles, ironability was possible and the polymer coating served as lubricant. The material presented in our research offers several advantages over preceding materials, including:

- Maximizing steel–polymer interface adhesion by appropriately selecting the polymer.
- Desired permeability can be implemented on the exterior surface, which can help in decoration.
- Formability can increase by modifying the mechanical properties of the polymer layers.
- Different design objectives are possible by altering the thickness of each layer.

This work investigated under which conditions a polymer-coated steel must be processed to produce food/beverage containers. It is the first time that two-layer polymer-coated steel has been investigated for can manufacturing. It reveals that polymer layers can survive the most critical process: ironing. If can manufacturers use this new material, they no longer need to place polymers by spraying inner and outer can sides, which thus avoids VOCs that are very harmful to the environment.

Any fractures of the delamination of polymer laminates can lead to subsequent corrosion and may spoil contents. Therefore, this new material is only useful if it remains undamaged in all the forming stages of can manufacturing. To generate the new surface, high pressures, strains and strain rates occur during ironing. Apart from the development of two theoretical models (UBM and ANN), friction, wear, and roughness analysis have been conducted on the material.

## 2. Materials and Methods

### 2.1. Material

**Figure 2:**
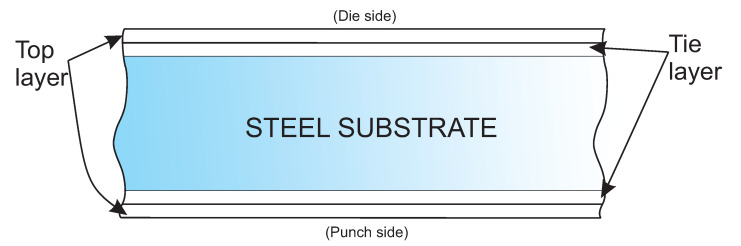
Illustration of the used material.

The material was provided by ArcelorMittal (Chicago, IL, USA). The innovative steel coated with two polymer layers on both the punch and die sides is illustrated in Figure 2.

The two layers are the tie layer, which is the layer that bonds the steel substrate to the top layer, externally, with a standard total thickness within the 12.5–35 μm range. Top layers provide mechanical strength and tie layers are intended for adhesion and anticorrosion properties. To precisely measure polymer layers, Wagner’s dis-indentation method can be used [9]. These polymeric layers can be adapted to any conditions and are made of PET which is more resistant than lacquer as a product barrier, bottom protection and wall denting. It also offers new decoration possibilities (white, clear or pigmented polymer).

**Figure 3:**
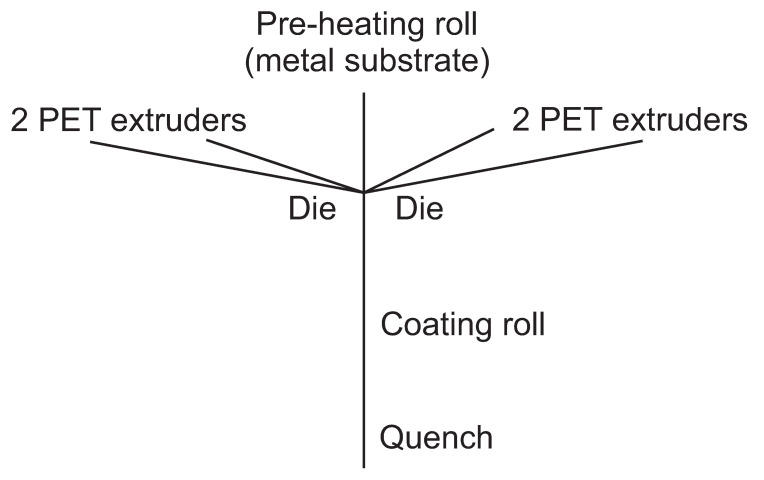
Extrusion coating process.

ArcelorMittal manufactures this new material by a compact and evolutive process using PET as a starting point instead of laminated film. The layer coatings of both sides are produced at the same time (Figure 3).

**Figure 4:**
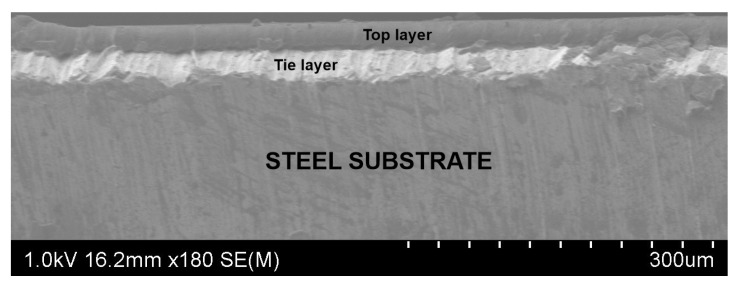
SEM image of the material.

A Hitachi SU-70 field emission scanning electron microscope (Japan) captured a SEM image of part of the material. The real thicknesses of both the top and tie polymeric layers is observed in Figure 4.

### 2.2. Experimental Procedure

**Figure 5:**
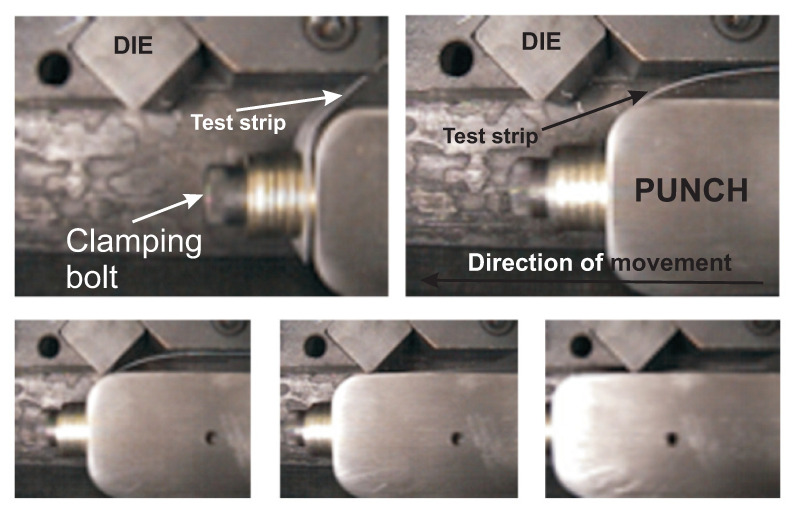
Five frames showing the exact moment when the ironing simulator irons a strip.

As mentioned in the Introduction, ironing is the most crucial process in can manufacturing. It is important to demonstrate that material survives this process. A strip ironing simulator was used for the experimental research. Figure 5 shows five frames indicating the exact moment the ironing simulator irons a strip. This device was developed at the University of Notre Dame. Basically, it has a punch where a metal strip can be attached by a bolt. Then with the help of a 22 kW motor, the punch moves to the left and forces the strip to pass through the small space between the punch and die, which is smaller than the workpiece’s total thickness. This movement reproduces the basic principle of the ironing process, which is the most critical one in can manufacturing given the high stresses involved. The maximum punch speed reached with this device is 2 m/s.

Under numerous testing conditions, the two-layered polymer material displayed excellent ironability. A design-of-experiments was prepared and the varied parameters included punch velocity, die angle, thickness reduction and die temperature. The two expected results of the experiments were: good ironing process or a bad result (one or two damaged polymer layers).

### 2.3. UBM Models

In order to accurately predict the possible outcomes of an ironing process using multilayered polymer steel coatings, a good theoretical model must be developed. Although the Finite Element Method (FEM) is the most widespread by researchers [8,10,11,12,13], it is hard to apply with predictive models. The Upper Bound Method (UBM) is a known methodology that accurately models real processes, and is preferred by some researchers [3,4,14].

With the UBM, it is assumed that the given material undergoes any deformations needed to achieve the final desired shape in a kinematically admissible flow field [15]. The principal issue encountered by using the UBM is the a priori displacement assumption needed, but this is usually be overcome by employing the values calculated from the slip line theory, from experimental data or by intuition.

For the present research, there were two possible outcomes: ironing successfully or ironing under a shaving condition. If the latter is present, the material is damaged as a result, and any of the following effects will also occur: the top polymer layer is only damaged, or both the top and tie polymer layers are damaged.

Two UBM models were developed for the two possible results: good ironing or shaving condition. Damage to the two polymer layers requires more power than that required to cause damage only on the external layer of a given polymer, and models include various assumptions used primarily for simplification purposes.

These assumptions are that the material is taken to be rigid and solids are completely plastic. However, it is interesting that a polymer behaves like these assumptions; the material rarely acts as entirely plastic. However, a certain number of shear planes is essential to enhance the accuracy of power estimations. Challen et al. [16] proposed the theory of an operational shear strength for the polymer, which would allow improved accuracy when applying the model of strength. It is described as:

(1) $$\bar{k}=\frac{1}{\gamma_{t}}\int_{0}^{\gamma_{t}}k\left(\gamma \right)d\gamma ,$$

where *k* is shear stress, $\gamma_{t}$ is shear strain, and $\bar{k}$ is shear strength. Polymer shear strength, $k_{i}$, is described as a portion of the whole piece shear strength, $k_{p}$, in both models presented in this work.

There is a unique friction factor associated with each interface. The punch-piece boundary is represented by $m_{1}$, the metal-tie layer boundary by $m_{2}$, the tie-top layers boundary by $m_{3}$ and, lastly, $m_{4}$ is the friction on the die-top layer boundary. The ironing process is ignored because tests have determined that the coating on the punch side of the sheet persists throughout this process.

#### 2.3.1. Model for Ironing

**Figure 6:**
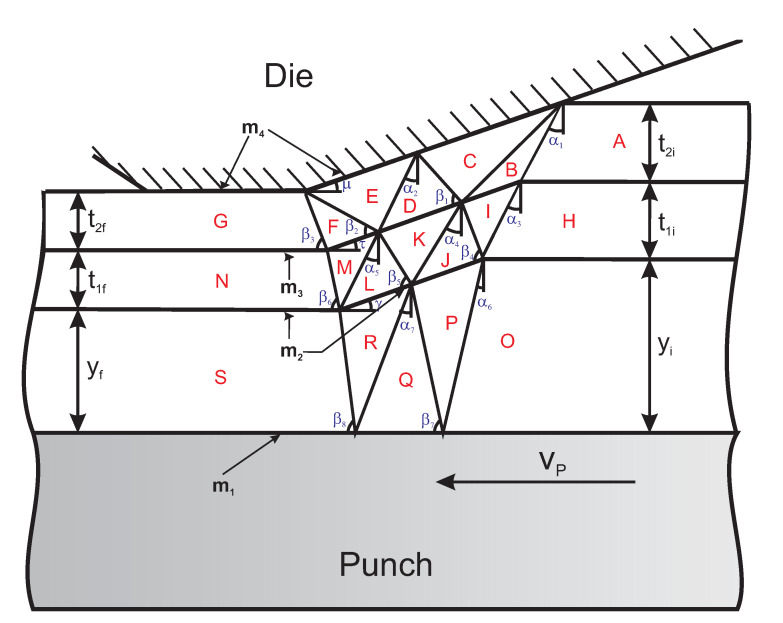
Illustration of the VDF for good ironing.

Figure 6 illustrates the velocity discontinuity field (VDF) associated with the successful ironing process involving the new coated steel. The Die-G plane is assumed to continue along the entire land length.

Angles $\alpha_{1}$–$\alpha_{7}$ and $\beta_{1}$–$\beta_{8}$ influence deformation planes, including the specified reduction and angles ϕ (die angle), μ, τ, and γ.

#### 2.3.2. Model for Shaving

**Figure 7:**
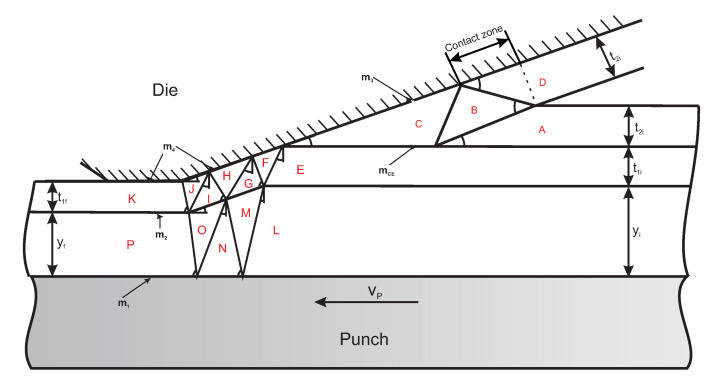
Illustration of the VDF for bad ironing.

Figure 7 presents the VDF associated with a bad ironing process.

The layer removed as result of shaving is region D, and its initial and final thicknesses are the same. An estimate was used for the interaction area between the die and this layer because the real area was very complicated to determine. With the approach presented by Wilson [17], the contact length between the die and region D is depicted in Figure 7.

### 2.4. Artificial Neural Network (ANN)

In order to accurately model the process, it was also modeled by an Artificial Intelligence (AI) technique by obtaining an ANN for this purpose.

**Figure 8:**
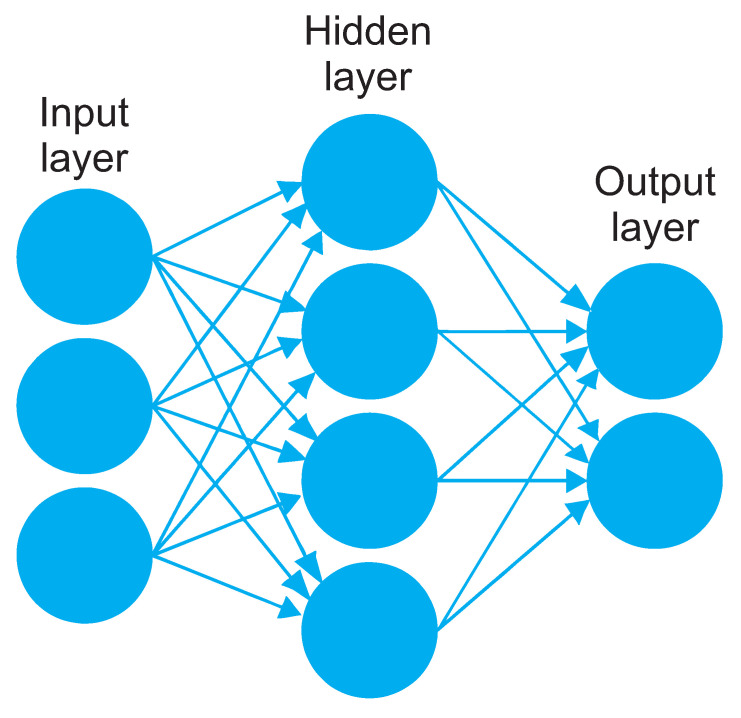
Typical ANN structure.

An ANN is a computational model that is practically designed to imitate the functioning of a natural network of neurons in order to carry out learning and problem-solving tasks, predictions and recognition, and all based on input data. It works similarly to natural networks; information is captured by sensors, basically electronic devices, sensors, etc. Input data are processed by a previously prepared neural network to finally draw conclusions, namely output data that serve to solve a specific problem. A typical ANN is shown in Figure 8.

The image represents a neural network with its respective components. Inputs that can be data or signals are on the left. These data enter the neurons on the input layer to be processed. Once processed, the neurons of the input layer send information to the next layer, in this case to the hidden layer. There is only one hidden layer in Figure 8, but the optimal ANN for a specific process can have more hidden layers. They also process information and send it to the output layer that will result in a solution.

So ANNs consist of neurons that can be configured and distributed in many ways depending on their final objective. A network can vary as to the number of neurons on the input layer, the number of hidden layers, their respective numbers of neurons on each layer and neurons on the output layer. Neurons are connected to them with different weights, which are modified in each learning cycle until an optimal value is reached.

The architecture and structure of an ANN largely depend on the type of function or task it will perform. In our case, a neural network was trained, structured and coded specifically for the ironing process in can manufacturing. In this way, someone can ensure that the network properly operates in a real environment.

The Deep Neural Networks concept came about as a result of using many hidden layers in networks (DNN) [18]. The ANN is trained once weights obtain the best possible value and then a very minor error.

There are basically two types of learning for ANNs: supervised and unsupervised. During supervised learning, examples of several problems are presented to the system along with the corresponding solution. System parameters are then adjusted to lower the value of a function of both the network configuration and the input/output variables whose value is a measure of the system’s adaptation to solve the problems that arise in the system during training.

During unsupervised learning, the system’s characteristics are modified by applying general heuristic rules without taking into account the specific characteristics of the universe in which patterns are generated.

The most widely used strategy to train ANNs is the algorithm known as Back Propagation (BP), which is a supervised learning procedure based on reducing the total square error at the network’s output.

During learning, patterns are placed on the input layer to propagate information to the output layer, which allows an error signal to be obtained for each processing element on the last layer. The resulting error signals are good feedback to the input layer. Based on the error signals, connection weights are then updated and the network converges to a configuration in which all the patterns in the training set are encoded.

The ANN was developed by the commercial software JustNN©. In order to perform ANN training, the selected algorithm was BP. Table 1 shows the variables employed in the ANN. The surface quality factor (SQF) is an output variable that was established to quantify how visually a surface differs from a perfect reference (with no damage). The range of possible values goes from 0 to 10, and this last value indicates that the surface is perfect. In this way, a surface with no damage will have an SQF of 10. The SQF will take a value of 0 for another surface where damage is seen on the two polymer layers.

**Figure 9:**
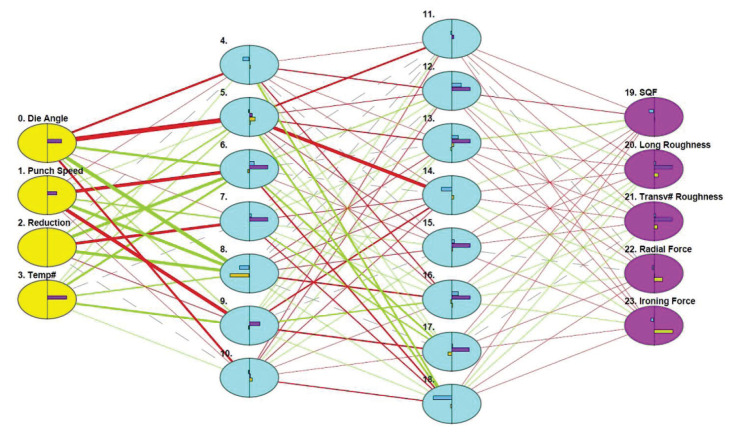
The ANN created for the ironing process. The four input variables are on the left, and the five output variables on the right. Blue columns are the two hidden layers. Different line thicknesses represent the connection weights.

Many different ANN configurations were tested with one, two, three, four and five hidden layers. At the end of the process, the optimal configuration was that with two hidden layers (Figure 9). To fully train this ANN, 76,316 cycles were required. It has four layers (input, two hidden layers, output), and are all connected through 342 connections. The final estimated error was very low, only $3\times 10^{-3}$.

## 3. Results

### 3.1. Theoretical Results

#### 3.1.1. UBM Models Results

The results vary depending on whether the successful ironing model or the shaving model was implemented. The deformation mode requires less power dissipation, and the UBM indicates that this rule will be followed by the current process. A comparison between the resulting curves for the ironing and shaving models indicated the preferred mode for a given set of conditions.

Whenever possible, a nondimensionalized form of the process input power was implemented as $P/kay_{i}v_{p}$; *P* is the total power required, *k* is strip shear strength, *a* is strip width, $y_{i}$ is the workpiece thickness at the beginning of the process, and $v_{p}$ is punch speed. The variable inserts allow for different process geometries to be tested.

**Figure 10:**
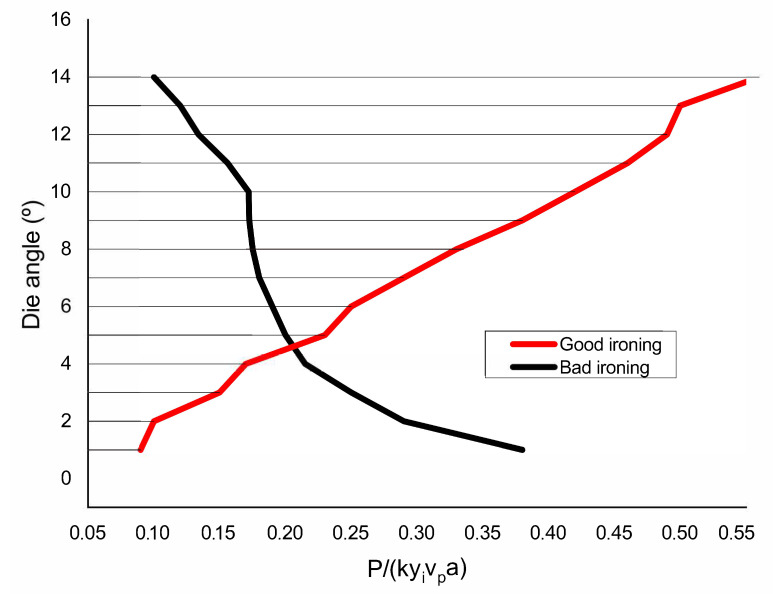
Optimized good and bad ironing curves.

A comparison between shaving and ironing conditions of die angle vs. optimal power curves are seen in Figure 10. Below angle $\phi \approx 4.6^{\circ }$, a good ironing process needs to use less power by the UBM, which makes it the favorite mode for these conditions. Above angle $\phi \approx 4.6^{\circ }$, shaving requires less power and is thus the favorite mode. From a formability standpoint, the critical angle, $\phi_{c}$, is very important because it influences the die geometries needed for the successful ironing of this new material.

Van der AA et al. [8] developed a model for ironing a one PET layer-coated steel by Leonov equations. They implemented these equations into a robust algorithm. The pressure accumulation on the polymer coating was a key parameter for the ironing process of the polymer-coated sheet metal, as well as the polymer’s strain rate dependence. Angles below $15^{\circ }$ resulted in good ironings. As our research demonstrated, optimal die angles were smaller if the metal was coated by two polymeric layers.

#### 3.1.2. ANN Results

The software used to generate the ANN can also provide interesting analysis data. The sensitivity analysis is defined as “the study of how the uncertainty in the output of a model can be apportioned to different sources of uncertainty in the model input” [19]. That is, a sensitivity analysis evaluates how an output variable reacts to changes in input variables. For an optimal output model of good ironing, Table 2 shows the sensitivity analysis values for each input variable.

The most important input variable in the model is die angle, as shown in Table 2. Few variations in this angle produce big differences in output variables. Punch velocity is also extremely important as it gains insight into which variable to focus on when manufacturing cans with this new material.

### 3.2. Experimental Results

#### 3.2.1. Surface Quality Factor

**Figure 11:**
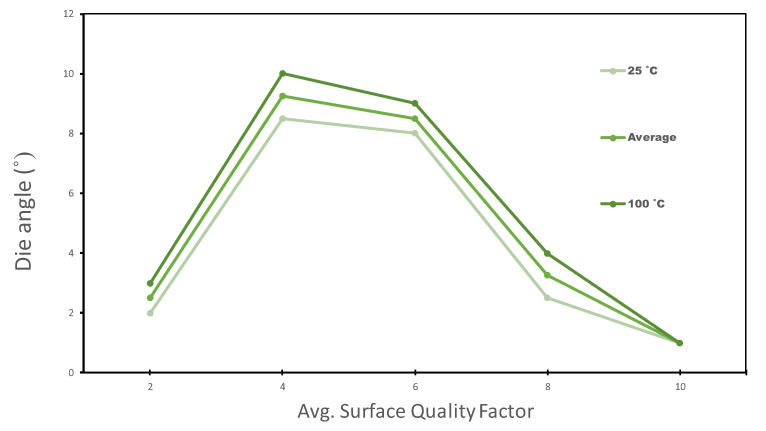
The average SQF vs. die angle. Ironed surfaces are adequate for can-decoration at values over 8.

The SQF shows how good the material surface is after the process. As indicated above, a value of 10 indicates that the surface is perfect, and decoration can be applied perfectly to it. A value of 0 means that the both top and tie layers were completely destroyed during the process. Figure 11 shows the SQF observed values versus die angle. As the die angle increased, the occurrence of shaving became more frequent, and the SQF had high values at angles close to $6^{\circ }$. The SQF also improved with high temperatures, as seen in Figure 11, but there was practically no difference.

Huang et al. [7] investigated the thermal effects on a one-layer polyester-coated steel, and found that the critical die angle at room temperature was $9^{\circ }$. The two-layer PET coated steel (our case) critical angle is $6^{\circ }$, because it has more polymeric layers.

#### 3.2.2. Roughness

**Figure 12:**
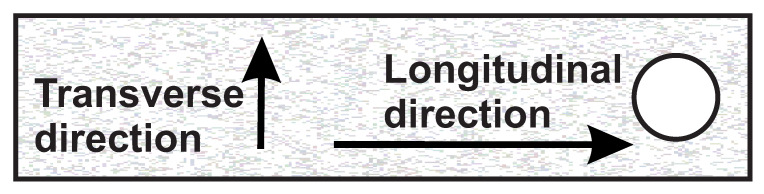
Roughness measurement directions in a strip.

**Figure 13:**
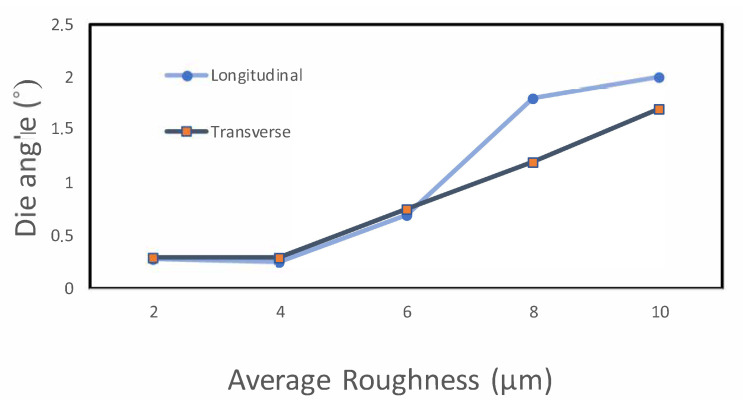
Die angle vs. roughness measured in both directions.

With the SJ-310 Portable Surface Roughness Device (MITUTOYO, Japan), measurements were taken on the surfaces of all the samples, and data on the average roughness in both the transverse and longitudinal directions were obtained [Figure 12]. For each surface, three measurements were taken in each direction, and the average was calculated. By looking at the roughness in Figure 13, we find a significant correlation between the longitudinal roughness and die angle (in the ironing direction) compared to the average roughness in the other direction. As Figure 13 depicts, roughness markedly decreased by going from $8^{\circ }$ to $6^{\circ }$, which evidences an improved polymer surface, and if we consider Figure 11 and Figure 13, both the $2^{\circ }$ and $4^{\circ }$ angles provide good results on the top surface.

**Figure 14:**
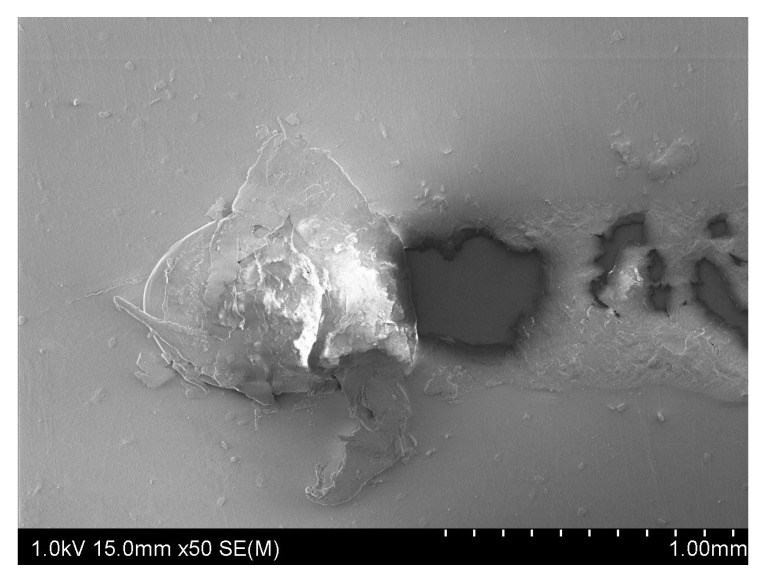
SEM image of a damaged zone on the top layer.

Figure 14 shows a SEM image of material removal on the top layer caused by a wrong ironing process. In the experiments conducted for this study, a bad result in the process did not result in removing the tie layer. If greater thickness reductions were investigated, these results could differ.

#### 3.2.3. Friction and Wear Volumes

The top layer can act as solid lubricant [20] during the forming processes involved in can manufacturing, especially in the ironing one, which was why a tribological analysis was run with the material with a linear tribometer (Ducom TR-282, Bohemia, NY, USA). To carry out the experiments, tests with a ball pressed against a plate surface were done to obtain the results of sliding wear on sample surfaces.

The coefficient of friction (COF) was obtained directly from the tribometer using a ball with a 10 mm. diameter. The conditions employed for the different tests are shown in Table 3. Two tests were carried out per condition.

**Figure 15:**
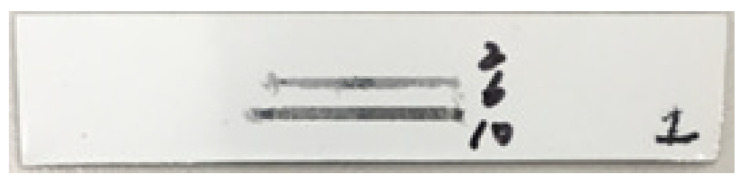
Wear tracks on the strip caused by forces of 2 N, 6 N and 10 N.

**Figure 16:**
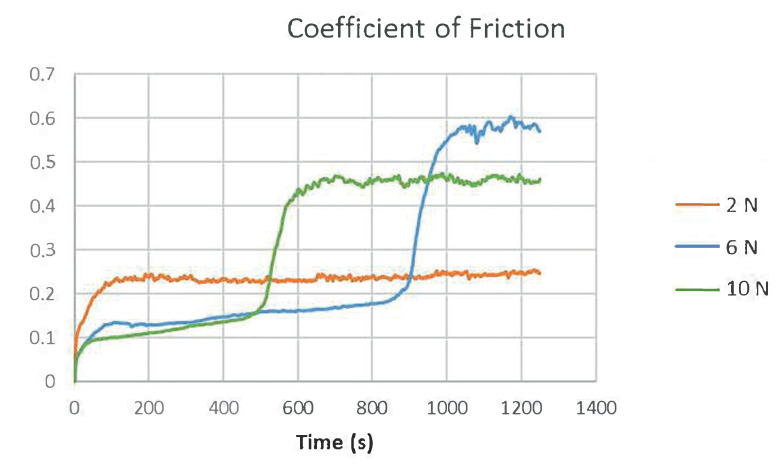
Friction coefficients over time with forces of 2 N, 6 N and 10 N.

Figure 15 shows the wear tracks caused by different loads on the material. An applied force of 10 N completely shaved the top layer. Friction coefficients (Figure 16) increases as soon as the coating layer wore out at higher loads. The 6 N load significantly increased the COF after 900 s. approximately, but then the COF reached its highest values.

**Figure 17:**
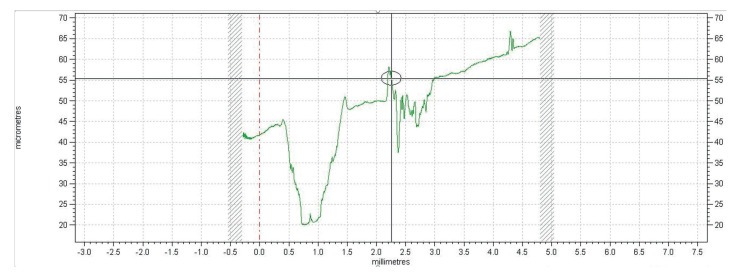
Wear profile tracks for the 10 N, 6 N, and 2 N forces.

The wear profiles for the considered loads are shown in Figure 17. The track length is 5 mm.

#### 3.2.4. Analysis of Variance (ANOVA)

The analysis of variance was performed mainly due to RA Fisher, whose works have greatly influenced modern statistics. It is used to analyze the data deriving from experiments to estimate and test hypotheses by considering the variances of samples, and from estimate and test hypotheses by considering sample means.

The ANOVA is perfect when we have three groups or more or conditions, and we wish to know if there is a significant difference between them, as with our process variables. Table 4 shows the ANOVA results for all four input variables. We can clearly see that the die angle is the most important factor for the quality of samples, with a marked difference compared to the other variables. The generated ANN also shows that the die angle is the most influential variable.

In Table 4 we see how important ironing speed is and better results were given by the process when working at higher speeds.

The final objective was to obtain a high SQF value. To do so it was necessary, as demonstrated, to work at high speeds and temperatures, as well as slight reductions.

## 4. Conclusions

The present research work demonstrates that food/beverages containers can be manufactured with this new material. For this to be possible, the most critical manufacturing process (ironing) must be carried out under certain conditions. Consequently, this methodology allows the production of containers and significantly reduced VOCs if this material is used.

The results shown in the previous section indicate a large correlation with the theoretical models (UBM and ANN). In fact thanks to the developed ANN, it is possible to modify the values of the reductions, layer thicknesses and die angles, and to immediately see the impact of these changes on the final quality of metal containers. This will certainly help packaging designers and manufacturers.

Regarding the influence of each input variable on final quality, the die proved to be the most important, and displayed good behavior when processing with angles below $6^{\circ }$. The other variables did no significantly influence the results.

The material friction and wear tests demonstrated that the top layer can act as a perfect solid lubricant. At the 2 N constant load, the COF value remained at 0.24 approximately, whereas higher loads increased this value to 0.5. The 6 N load significantly increased the COF after 900 s, while the 10 N load increased the COF after 500 s.

This new material is able to withstand the major deformations that occur during ironing. Consequently, it is suitable for food packaging manufacturing.

## Figures and Tables

**Table 1:** Input and output variables of the ANN.

Input Variables	Units	Output Variables	Units
		Surface Quality	SQF
Die angle	°	Longitudinal roughness	um
Punch velocity	m/s	Transversal roughness	um
Reduction	%	Radial force	N
Temperature	°C	Ironing force	N

**Table 2:** Sensitivity analysis results for the input variables.

Input Variables	Units	Sensitivity
Die angle	°	0.132
Punch velocity	m/s	0.063
Reduction	%	0.005
Temperature	${}^{\circ }$C	0.001

**Table 3:** Selected conditions for wear tests.

Variables	Values		
Loads (FN)	2 N	6 N	10 N
Frequency (f)	2 Hz		
Sliding stroke	10 mm		
Sliding distance (cycle)	500 m	(in 50,000 cycles)	

**Table 4:** The ANOVA results.

Input Variables	Sum of Squares
Die angle	423.96
Punch velocity	15.20
Reduction	11.43
Temperature	16.32

## Data Availability

Not applicable.
